# Infectious Disease: Meaner MRSAs

**Published:** 2006-01

**Authors:** Carol Potera

Most methicillin-resistant *Staphylococcus aureus* (MRSA) infections are contracted in hospitals and other health care facilities. Antibiotic use, patients’ weakened immune systems, close contact among people, and open wounds all make hospitals prime breeding grounds for these bugs. But community-acquired MRSA strains, which attack healthy individuals with seemingly normal immune systems, are becoming more prevalent. A recent comparison of representative strains of hospital- and community-acquired MRSAs now suggests that the latter are more virulent and that they excel at escaping destruction by white blood cells.

Infectious disease experts suspected that community-acquired strains can overcome a healthy immune system because they operate differently than those acquired from hospital or health care settings. Microbiologist Frank DeLeo of the National Institute of Allergy and Infectious Diseases’ Rocky Mountain Laboratories led a multi-institutional team of researchers in comparing the two types. In studies described in the 15 September 2005 issue of *The Journal of Immunology*, they evaluated the potency of three community-acquired MRSA strains (MW2, LAC, and MnCop) and two hospital-acquired strains (MRSA252 and COL).

Healthy adult mice were injected with each strain. All the mice infected with community-acquired strains became ill, and several died. None of the mice infected with the hospital-acquired strains died, and only one mouse became ill. Then the MRSA strains were mixed with human neutrophils (white blood cells), the body’s first line of defense against bacterial invasion, which kill bacteria by producing hydrogen peroxide and other toxic oxygen metabolites. After half an hour, the community-acquired strains survived neutrophil destruction better than the hospital-acquired ones. After six hours, the community-acquired strains had begun rupturing the neutrophils and were actually growing.

Next the researchers used micro-arrays to uncover genes that differed during interaction with neutrophils. Not surprisingly, genes that encode virulence factors, toxin production, and stress responses were induced in all the MRSA strains. However, about two dozen genes that encode surface or secreted proteins of unknown function were upregulated only in the community-acquired strains. Gene knockout experiments are under way to identify whether these genes contribute to neutrophil killing. The researchers are also exploring how the community-acquired strains withstand neutrophils’ toxic compounds.

The findings suggest that community- and hospital-acquired MRSA strains differ broadly in their biology and genetics. Will this new information help physicians on the front lines who are fighting MRSA infections? “[The findings] do not have immediate therapeutic implications, but maybe down the line therapies will be developed based on such findings,” says Henry Chambers, an infectious disease physician at the University of California, San Francisco, School of Medicine.

## Figures and Tables

**Figure:**
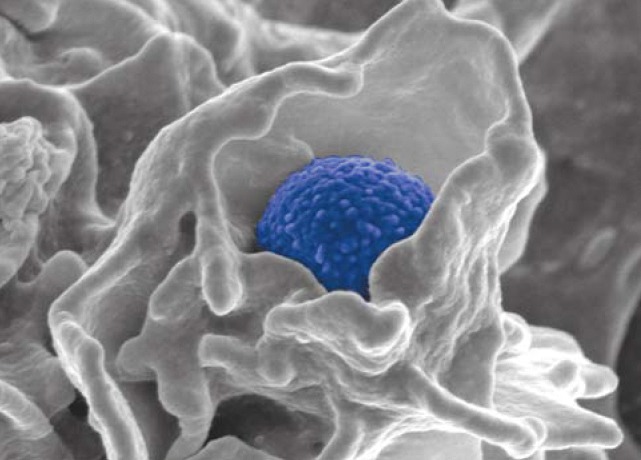
One bad bug. Community-acquired methicillin-resistant *S. aureus* (in blue) overcomes the immune system by destroying neutrophils, thus breaching the body’s first line of defense.

